# Structure and Function of ABCG2-Rich Extracellular Vesicles Mediating Multidrug Resistance

**DOI:** 10.1371/journal.pone.0016007

**Published:** 2011-01-24

**Authors:** Vicky Goler-Baron, Yehuda G. Assaraf

**Affiliations:** The Fred Wyszkowski Cancer Research Laboratory, Department of Biology, Technion-Israel Institute of Technology, Haifa, Israel; Université de Toulouse, France

## Abstract

Multidrug resistance (MDR) is a major impediment to curative cancer chemotherapy. The ATP-Binding Cassette transporters ABCG2, ABCB1 and ABCC2 form a unique defense network against multiple structurally and functionally distinct chemotherapeutics, thereby resulting in MDR. Thus, deciphering novel mechanisms of MDR and their overcoming is a major goal of cancer research. Recently we have shown that overexpression of ABCG2 in the membrane of novel extracellular vesicles (EVs) in breast cancer cells results in mitoxantrone resistance due to its dramatic sequestration in EVs. However, nothing is known about EVs structure, biogenesis and their ability to concentrate multiple antitumor agents. To this end, we here found that EVs are structural and functional homologues of bile canaliculi, are apically localized, sealed structures reinforced by an actin-based cytoskeleton and secluded from the extracellular milieu by the tight junction proteins occludin and ZO-1. Apart from ABCG2, ABCB1 and ABCC2 were also selectively targeted to the membrane of EVs. Moreover, Ezrin-Radixin-Moesin protein complex selectively localized to the border of the EVs membrane, suggesting a key role for the tethering of MDR pumps to the actin cytoskeleton. The ability of EVs to concentrate and sequester different antitumor drugs was also explored. Taking advantage of the endogenous fluorescence of anticancer drugs, we found that EVs-forming breast cancer cells display high level resistance to topotecan, imidazoacridinones and methotrexate via efficient intravesicular drug concentration hence sequestering them away from their cellular targets. Thus, we identified a new modality of anticancer drug compartmentalization and resistance in which multiple chemotherapeutics are actively pumped from the cytoplasm and highly concentrated within the lumen of EVs via a network of MDR transporters differentially targeted to the EVs membrane. We propose a composite model for the structure and function of MDR pump-rich EVs in cancer cells and their ability to confer multiple anticancer drug resistance.

## Introduction

The frequent emergence of drug resistance phenomena to structurally and functionally unrelated anticancer drugs known as multidrug resistance (MDR) continues to be a major impediment to curative cancer chemotherapy [1], [2], [3], [4], [5], [6]. Members of the ATP-Binding Cassette (ABC) superfamily of transporters including ABCB1 (P-gp), ABCC1 (MRP1) and ABCG2 (BCRP) function as ATP-dependent MDR efflux transporters. These multidrug extrusion pumps form a unique defense network against multiple chemotherapeutic drugs, as well as endogenous and exogenous cellular toxicants. We have recently found that in mitoxantrone (MR)-resistant MCF-7 breast cancer cells (MCF-7/MR) [7], ABCG2 is overexpressed and confined to cell-cell attachment zones, where ABCG2-rich extracellular vesicles (EVs) are formed [8]. Shared by neighbor cells, these EVs display a 1000-fold intravesicular concentration of MR when compared to its concentration in the culture medium, thereby resulting in MR resistance. Moreover, inhibition of ABCG2 transport activity with the specific transport inhibitors Ko143 and fumitremorgin C (FTC) abolished intravesicular MR accumulation, hence resulting in restoration of drug sensitivity.

In spite of the important implications of these drug-concentrating EVs for cancer chemotherapy, nothing is known about their structure, biogenesis and ability to sequester multiple anticancer drugs. Towards this end, we here explored the possible association of cytoskeletal components characteristic of polarized epithelia including tight junction (TJ) proteins, actin and microtubule filaments as well as Ezrin-Radixin-Moesin (ERM) complex proteins with the membrane of EVs. TJ proteins have three mutually exclusive functions; a fence function which differentiates between proteins of the apical and basolateral membranes, a gate function which controls the paracellular passage of ions and solutes in-between epithelial and endothelial cells, as well as a bridge function which facilitates the communication between neighboring cells [9], [10]. Proteins of the ERM complex are closely related polypeptides linking actin filaments to the plasma membrane either directly via binding to the cytoplasmic tail of membrane proteins, or indirectly via scaffold proteins attached to membrane proteins [11].

Here we show that EVs are apically localized and reinforced by cytoskeletal proteins. We provide evidence for the role of TJ proteins including occludin and ZO-1 in the formation of sealed EVs that are secluded from the extracellular milieu. We further demonstrate that apart from ABCG2, the membrane of EVs also contains the major MDR efflux transporters ABCB1 and ABCC2, thereby highly concentrating multiple anticancer drugs in the lumen of EVs. Furthermore, the ERM protein complex was found to be selectively targeted to the EV membrane, suggesting a functional role in the cross-linking of these MDR pumps to the actin cytoskeleton. Hence, a new modality of MDR is described here in which ABC transporters that are selectively sorted out to the membrane of EVs, actively pump and sequester multiple anticancer drugs within the EV lumen, thereby resulting in a marked chemoresistance.

## Results

### TJ proteins are involved in the formation of EVs that mediate MDR

**Figure 1:**
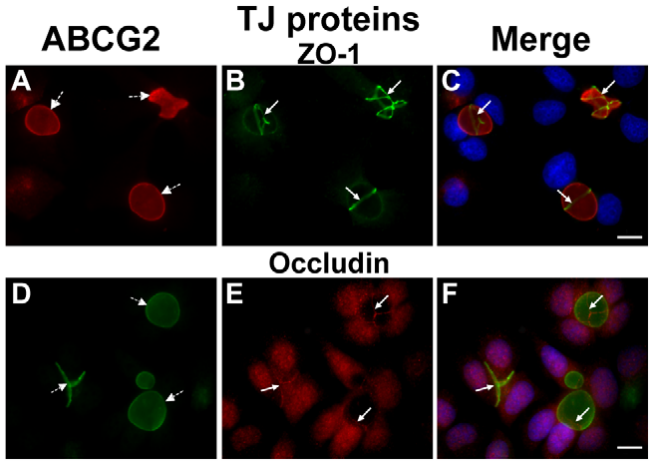
Specific targeting of TJ proteins ZO-1 and occludin to EVs in MCF-7/MR cells. MCF-7/MR cells were grown for 7 days to allow for the formation of EVs, fixed and co-reacted with monoclonal antibodies to ABCG2 (A, BXP-53; D, BXP-21), ZO-1 (B) and occludin (E). Panels C and F are merged photographs of A and B as well as D and E, respectively. *White arrows* denote the localization of TJ proteins, whereas *dashed arrows* represent EVs. Cells were examined with a Zeiss inverted Cell-Observer microscope at a magnification of ×630. The merged images, including nuclei stained with DAPI, were generated using Cell-Observer software to illustrate the three-dimensional architecture of EVs. Throughout the entire study the bar denotes10µm.

Previously we have shown that ABCG2-rich EVs of breast cancer cells concentrate riboflavin and MR by factors of 560- and 1000-fold, respectively, when compared to their concentration in the extracellular medium [8], [12]. To identify proteins that may contribute to the formation of these ostensibly sealed EVs, immunofluorescence microscopy was performed using antibodies specific to TJ proteins; this included occludin as a major TJ-forming protein which mediates cell-cell adhesion and ZO-1 as a representative TJ-associated protein that crosslinks TJs to the actin-cytoskeleton [13], [14]. MCF-7/MR cells were co-stained with antibodies to ABCG2 which served as a specific biomarker for EVs, as well as with ZO-1 (**Figure 1A–1C**) and occludin (**Figure 1D–1F**). Both ZO-1 and occludin displayed intense localization at cell-cell attachment zones, albeit occludin also had some cellular staining. These TJ proteins surrounded ABCG2-rich EVs, hence forming intense ring structures precisely at the border of EVs-forming cells. When a single EV is formed by two attached cells, one belt-like structure was located between these neighboring cells. Other EVs had several belt-like structures around them, indicating that three or more attached cells participated in EV formation (**Figure 1**). A small fraction (∼8.5%) of vesicular structures was found to be positive for ABCG2 but showed no discernible staining for ZO-1 and appeared to be intracellular (i.e. vacuoles; data not shown). Parental MCF-7 cells (ATCC number HTB-22), which are characterized by very low ABCG2 protein levels [8] and rarely form EVs (which are much smaller than in MCF-7/MR cells), exhibited consistent TJ localization to the EVs membrane (**Supplementary Figure S1**).

### EVs are formed at the apical pole of MCF-7/MR cells

**Figure 2:**
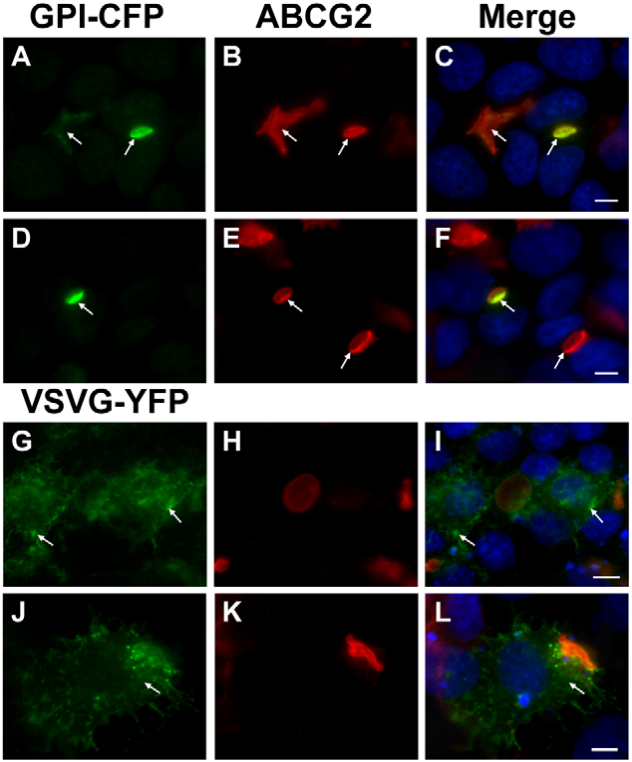
Apical localization of EVs in MCF-7/MR cells. Cells were transiently transfected with GPI-CFP and VSVG-YFP constructs (green fluorescence), reacted with anti-ABCG2 antibody BXP-53 (red fluorescence) and DAPI and analyzed using Zeiss inverted Cell-Observer microscope at a magnification of ×630. *Arrows* denote the subcellular localization of GPI-CFP (A–F) and VSVG-YFP (G–L).

During the current study we observed a strong similarity between the EVs that form in breast cancer cells and bile canaliculi, previously described in polarized WIF-B9 [15] and HepG2 [16] cells. We hence theorized that EVs are apically localized. To test this hypothesis, we ectopically overexpressed GPI-CFP and VSVG-YFP constructs which are harboring established apical and basolateral markers proteins, respectively [17], [18]. Expectedly, ABCG2, an established EV membrane marker co-localized with the apical GPI marker (**Figure 2A–2F**); in contrast, the basolateral marker VSVG was equally distributed in the cell membrane and did not display an exclusive co-localization with ABCG2, an EV membrane marker (**Figure 2G–2L**).

#### Selective targeting of MDR transporters to the membrane of EVs

**Figure 3:**
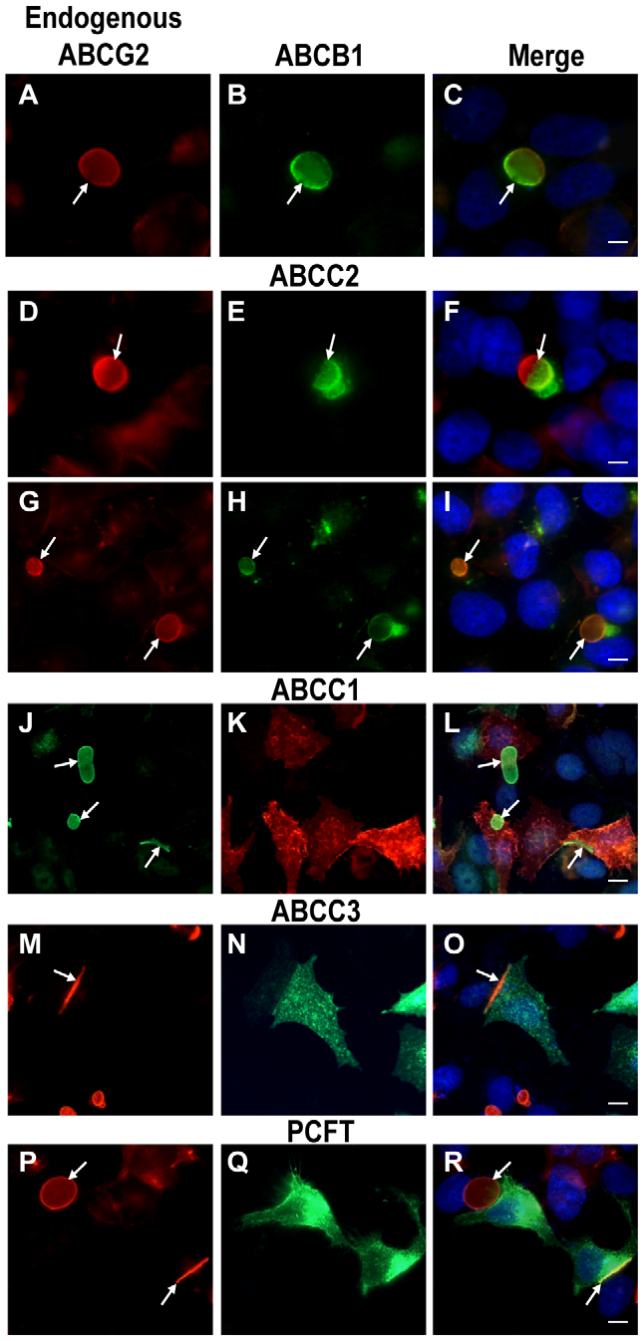
Specific targeting of transiently transfected ABCB1 and ABCC2 to the EVs membrane in MCF-7/MR cells. Cells were transfected with ABC transporters as described in Materials and Methods, and then studied by immunofluorescence microscopy. Shown are the subcellular localizations of ABCB1 (A–C), ABCC1 (J–L), ABCC2 (D–I), ABCC3 (M–O), and PCFT (P–R), when compared to that of ABCG2. ABCG2 was followed using the monoclonal antibodies BXP-21 (J) or BXP-53 (A, D, G, M and P). Ectopically expressed proteins are indicated on the top of each panel. Cells were examined using either a Leica (×400) or the Cell-Observer (×630) microscopes. *Arrows* denote the location of ABCG2-rich premature and mature EVs.

Endogenous ABCG2 is overexpressed in MCF-7/MR cells and is targeted differentially to the membrane of EVs [8]. However, in MCF-7/MR cells which are not part of a colony and/or that do not participate in the formation of EVs, ABCG2 typically localizes at the perinuclear ER area (**Supplementary Figure S2 G–I**, continuous arrows). Based on the localization pattern of TJ proteins to cell-cell contact sites of EVs-forming cells (**Figure 1** **and supplementary Figure S1**), we hypothesized that there may be a physical separation of the EV membrane from the cell membrane. To examine this hypothesis we first stably overexpressed an ABCG2-EGFP construct in parental MCF-7 and MCF-7/MR cells. Ectopic overexpression of ABCG2-EGFP resulted in differential transporter targeting in parental and drug resistant cells; in parental MCF-7 cells, which infrequently form EVs, ABCG2-EGFP localized primarily at the cell membrane (**Supplementary Figure S2 A–C**). However, when EVs infrequently formed in MCF-7 cells, ABCG2-EGFP was targeted to the EVs membrane. In contrast, in MCF-7/MR cells, ABCG2-EGFP was targeted exclusively to the membrane of EVs and cell-cell attachment zones (**Supplementary Figure S2 D–F**). Thus, we focused our further studies on MCF-7/MR cells in an attempt to explore the possible differential sorting of various MDR efflux transporters of the ABC superfamily including ABCB1, ABCC1, ABCC2 and ABCC3 (i.e. P-gp, MRP1, MRP2 and MRP3, respectively) to the EV membrane. These ATP-driven MDR efflux transporters differ markedly in substrate specificity, tissue distribution and intracellular localization [1], [4], [5], [6]. Previously we have shown that ABCC2 and ABCC3 levels are undetectable in MCF-7/MR cells, whereas low levels of ABCC1 were present [19]. To determine the targeting specificity of these ABC transporters to the EV membrane, we introduced these MDR transporter genes into MCF-7/MR cells and determined their sub-cellular localization by immunofluorescence microscopy. ABCB1 and ABCC2 were specifically targeted to the EV membrane and to cell-cell attachment zones, hence perfectly co-localizing with ABCG2 (**Figure 3A–3C and 3D–3I**, respectively). Analysis of ABCB1- and ABCC2-transfectant cells revealed that some EVs displayed a unique transporter co-localization pattern with ABCG2 and the exogenously transfected transporter solely present in one half of the EV membrane (**Figure 3A–3C and 3D–3F** *arrows*), whereas other EVs exhibited an equally distributed co-localization pattern (**Figure 3G–3I**). In contrast, in ABCC1- (**Figure 2J** **–** **3L**) and ABCC3-transfected (**Figure 3M–3O**) MCF-7/MR cells, these MDR transporters were equally targeted to the cell membrane. The proton-coupled folate transporter (PCFT/SLC46A1), which is representative of various proton-coupled low pH carriers, that mediate intestinal absorption of various essential nutrients [20], [21], [22], was further examined. Consistent with previous findings [22], [23], PCFT, the dominant intestinal folate transporter [22], was targeted to the entire cell membrane of MCF-7/MR cells, thus no co-localization of PCFT and ABCG2 was found in the EVs membrane (**Figure 3P–3R**). The subcellular localization of Na^+^/K^+^ ATPase was also determined using immunohistochemistry and confocal laser microscopy, as this central ATP-driven cation pump is ubiquitously expressed at high levels and targeted to the basolateral membrane of secretory epithelial cells including the intestine, glands and kidney [24]. Na^+^/K^+^ ATPase was targeted to the cell membrane of MCF-7/MR cells, but not to the membrane of EVs (**Supplementary Figure S3 and supplementary Video S2**).

### The ERM protein complex selectively localizes to the border of the EV membrane

**Figure 4:**
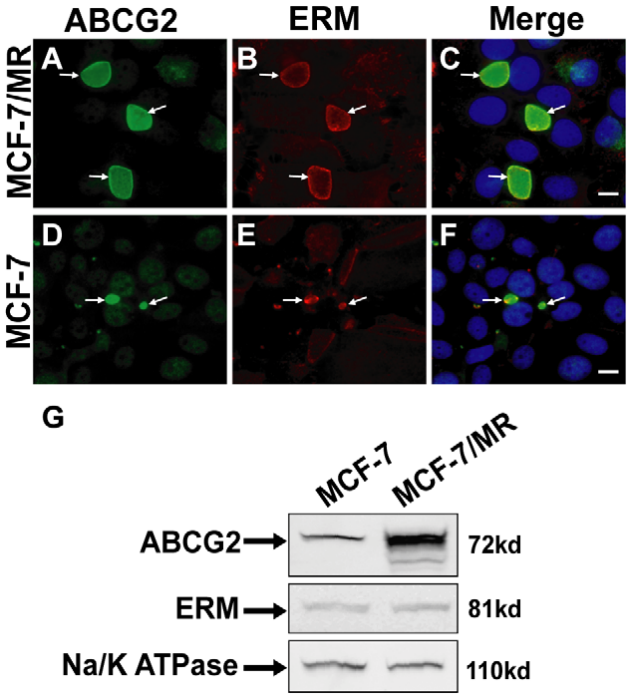
Subcellular localization of the ERM protein complex in MCF-7 and MCF-7/MR cells. MCF-7/MR (A–C) and MCF-7 cells (D-F) were co-reacted with antibodies to ABCG2 (BXP-21, A and D) and ERM (B and E). Random colonies were analyzed under Zeiss inverted Cell-Observer microscope at a magnification of ×630. Western blot analysis of ERM levels in MCF-7 and MCF-7/MR cells (G).

Based on the above findings we further hypothesized that the EV membrane residents ABCG2, ABCB1 and ABCC2 may be anchored to the cytoskeleton via proteins of the ERM complex [11], [25]. To this end, immunofluorescence microscopy was performed using monoclonal antibodies to ERM proteins and ABCG2 (**Figure 4**). ERM proteins exclusively co-localized with ABCG2 at the EV membrane in both MCF-7/MR (**Figure 4A–4C**) and their parental MCF-7 cells (**Figure 4D–4F**). The mean percentage of co-localization of ERM proteins and ABCG2 in EVs was determined; analysis of 4,000 MCF-7/MR cells harboring 860 vesicles revealed 100% co-localization of ERM proteins and ABCG2 in the EV membrane. Western blot analysis of ERM protein expression revealed that in spite of the spectrum-wide different patterns of EVs shapes and sizes in MCF-7 and MCF7/MR cells, ERM protein localization (**Figure 4A–4F**) and expression levels (**Figure 4G**) were identical in both cell lines.

### Cytoskeletal proteins, actin and microtubules reinforce the EVs structur

**Figure 5:**
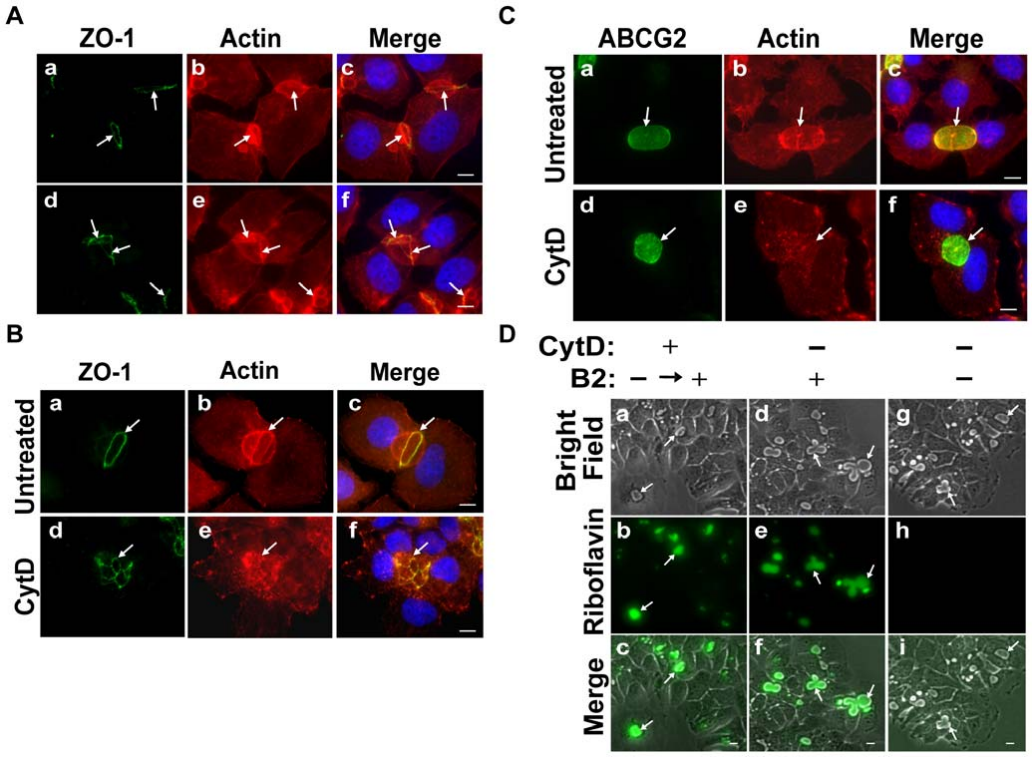
Localization of F-actin in MCF-7/MR cells and inhibition of actin polymerization by CytD. (A) Cells grown for 7 days on glass cover slips were fixed, permeabilized with Triton-X-100 and reacted with anti-ZO-1 antibody (a and d) and with rhodamine phalloidin (b and e) to follow F-actin. The localization of actin and ZO-1 at the EVs surface is shown for EVs formed between two (a–c) and/or multiple attached cells (d–f). *Arrows* denote the localization of cell-cell attachment zones (belt-like structures). (B) Cells were either untreated (a–c) or treated with CytD (10 µg/ml) for 30 min at 37°C (d–f), washed and reacted as in panel A, to visualize ZO-1 (a and d) and F-actin (b and e). (C) Cells were treated as described in panel B and then stained for ABCG2 (a and d) and F-actin (b and e). (D) Cells were grown in riboflavin (B2)-deficient medium for 48 hr prior to CytD treatment to avoid riboflavin accumulation in EVs. Cells were then washed and transferred to riboflavin-containing medium for an additional 24 hr to examine the riboflavin accumulation capacity (a–c). Untreated cells grown continuously in medium containing (d–f) or lacking riboflavin (g–i) served as controls. *Arrows* denote the location of EVs. Cells were analyzed using a Zeiss inverted Cell-Observer microscope at a magnification of ×630 (A–C) or ×200 (D). The merged images including DAPI staining (panels A–C), were generated using Cell-Observer software.

To characterize cytoskeletal proteins that may reinforce the oval shape of EVs, we studied the subcellular localization pattern of F-actin in relation to EVs in MCF-7/MR cells (**Figure 5A**). In addition to its expected localization to cell-cell attachment zones and its co-localization with ZO-1, F-actin was highly targeted to the periphery of the EVs membrane, thus forming an actin-rich brush border characteristic of the apical side of polarized epithelia [26]. Furthermore, confocal microscopy confirmed that vesicular F-actin was radiating from the cytoplasmic face of vesicle-forming cells towards the vesicular membrane (**Supplementary Video S1**). Hence, the possibility that F-actin resides in the lumen of EV was ruled out.

To examine the possible roles of actin polymerization in the biogenesis of EVs and endocytosis of transmembrane proteins, MCF-7/MR cells were treated with cytochalasin D (CytD), a potent mycotoxin inhibitor of actin polymerization which disrupts microfilaments [27]. Upon CytD treatment, immunofluorescence microscopy revealed that actin predominantly appeared as small aggregates rather than actin filaments, and cell membrane localization of actin was markedly reduced (**Figure 5B, d–f**). However, ZO-1 was retained in cell-cell attachment zones, hence implying that actin is presumably not the sole cytoskeletal protein supporting vesicular structure. This pulse treatment with CytD had no effect on ABCG2 localization to the EVs membrane (**Figure 5C**). Consistently, CytD had no deleterious effect on the highly concentrative transport function of vesicular ABCG2 as revealed by the intense riboflavin fluorescence accumulated within the lumen of EVs in MCF-7/MR cells (**Figure 5D**).

**Figure 6:**
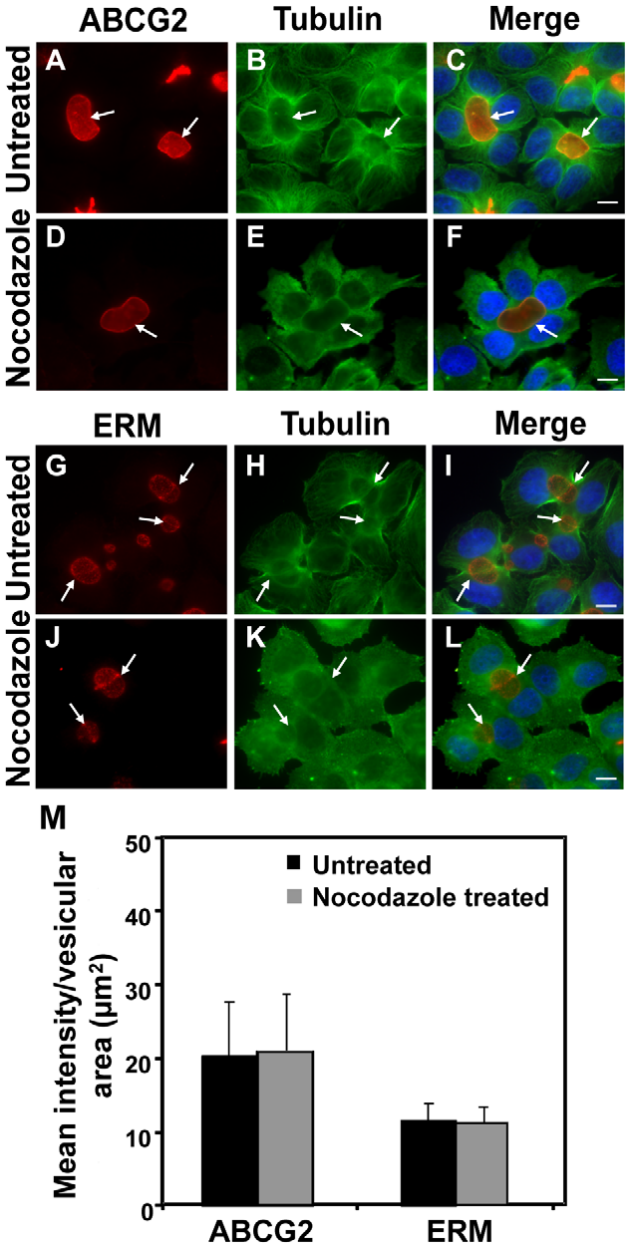
Involvement of microtubules in the formation of EVs in MCF-7/MR cells and inhibition of microtubule polymerization by nocodazole. MCF-7/MR cells were either untreated (A–C and G–I) or treated with nocodazole (33 µM) for 1 hr at 37°C (D–F and J–L). Microtubules were visualized using mouse anti β-tubulin antibody followed by incubation with FITC-conjugated donkey anti-mouse IgG (B, E, H and K). Cells were co-reacted with antibodies to ABCG2 (BXP-53, A and D) or ERM (G and J), processed and analyzed as in Fig. 5 legend. Quantification of ABCG2 and ERM protein expression on the surface of EVs prior to and following treatment with nocodazole (M); random fields stained as in upper panels were photographed using the same exposure conditions for untreated and nocodazole-treated cells. The surface area of EVs and its relative fluorescence intensity were estimated using the AxioVision program. The fluorescence at the EVs surface indicated protein levels at the EVs membrane. A total of 100 EVs were analyzed for each examined protein. Bars represent SD.

Based on our previous observation that EVs are dynamic structures that are easily disrupted upon standard trypsinization, we theorized that inhibition of microtubule polymerization may interfere with EVs formation and/or with vesicular trafficking of transmembrane proteins including ABCG2. To test this hypothesis, we first examined the distribution pattern of microtubules in relation to EVs location. In contrast to the localization pattern of actin filaments, there was no significant accumulation of microtubules around EVs (**Figure 6A–6C**). Microtubules were highly concentrated in the vicinity of EVs and appeared as radiating out both towards the cell membrane and the nucleus. We therefore used nocodazole, an established inhibitor of microtubule polymerization, which abolishes apical trafficking of proteins [16], [28]. Treatment with nocodazole resulted in disruption of the fine microtubular network that was observed in untreated cells. However, localization of the EV biomarkers ABCG2 and ERM complex proteins was not significantly affected (**Figure 6D–6F; 6J–6L** respectively). We hence quantified the co-localization of ABCG2 and ERM proteins to the surface of EVs prior to, and following treatment with nocodazole (**Figure 6 M**). Mean fluorescence intensity of ABCG2 and ERM proteins that localize to EVs (normalized to vesicular area) was not affected by nocodazole treatment. The relatively large SD observed with ABCG2 analysis is due to the fact that MCF-7/MR cells were established by pulse exposure to MR, thus resulting in a heterogeneous cell population with a large Gaussian distribution of ABCG2 expression (**Supplementary Figure S4**) and function [19]. Indeed, flow cytometric analysis of surface expression of ABCG2 in viable cells confirmed the large Gaussian distribution of ABCG2 expression in MCF-7/MR cells and ABCG2-overexpressing A549/K1.5 non-small lung cancer cells **(Supplementary Figure S4**).

#### Formation of EVs in various human malignant tumor cell lines

**Figure 7:**
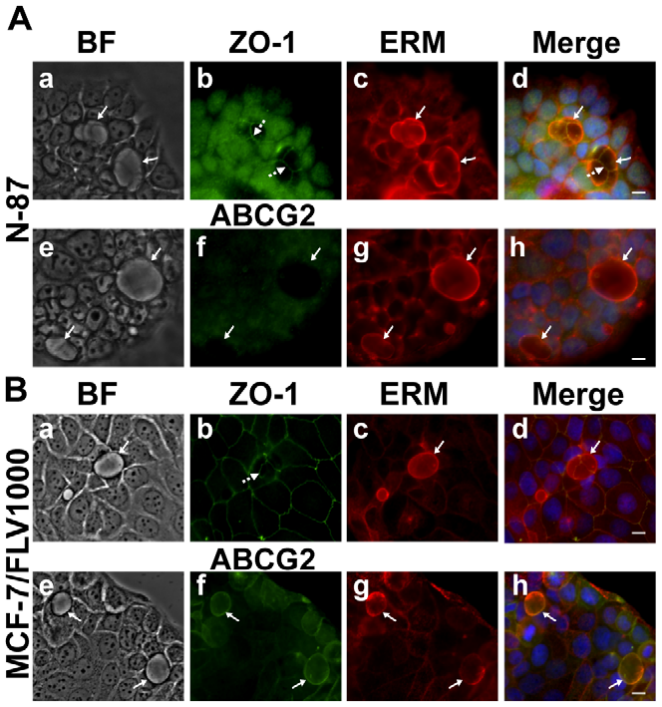
Formation of EVs in various human malignant tumor cell lines. Human gastric carcinoma N-87 (A) and flavopiridol-resistant breast cancer MCF-7/FLV1000 cells (B) were fixed and co-reacted with antibodies to ABCG2 (f), ZO-1 (b) and ERM complex (c and g) as detailed in Materials and Methods. Cells were then examined with a Zeiss inverted Cell-Observer microscope at a magnification of ×400. The merged images including nuclei stained with DAPI were generated using Cell-Observer software. BF indicates bright field mode. *Continuous arrows* denote the localization of EVs markers (either ABCG2 or ERM complex), whereas *dashed arrows* indicate TJ localization.

The existence of EVs in tumor cells other than the MCF-7/MR breast cancer subline was previously identified in our recent paper with the ABCG2-overexpressing non-small lung cancer A549/K1.5 cells [12]. Thus, we next explored the possible formation of EVs in additional human malignant tumor cell lines including gastric carcinoma N-87 cells that lack ABCG2 expression and flavopiridol-resistant breast cancer MCF-7/FLV1000 cells with ABCG2 overexpression. Strikingly, these carcinoma cell lines which are of distinct cell lineage formed EVs that were identified using vesicular markers; in N-87 cells, EVs were identified using an antibody to the ERM complex (**Figure 7A**), whereas EVs in MCF-7/FLV1000 cells were identified with antibodies to both ABCG2 and ERM complex (**Figure 7B**). Moreover, EVs from both cell lines exhibited a structure that is sealed by tight junctions (**Figure 7b in A and B**). Interestingly, A549/K1.5 [12] and MCF-7/FLV1000 cells also exhibited an ABCG2 function similar to MCF-7/MR cells; they showed a marked accumulation of riboflavin in EVs, whereas EVs from N-87 cells were completely devoid of riboflavin accumulation, which is consistent with the undetectable levels of ABCG2 in these cells (data not shown).

### EVs mediate resistance to multiple anticancer drugs

**Figure 8:**
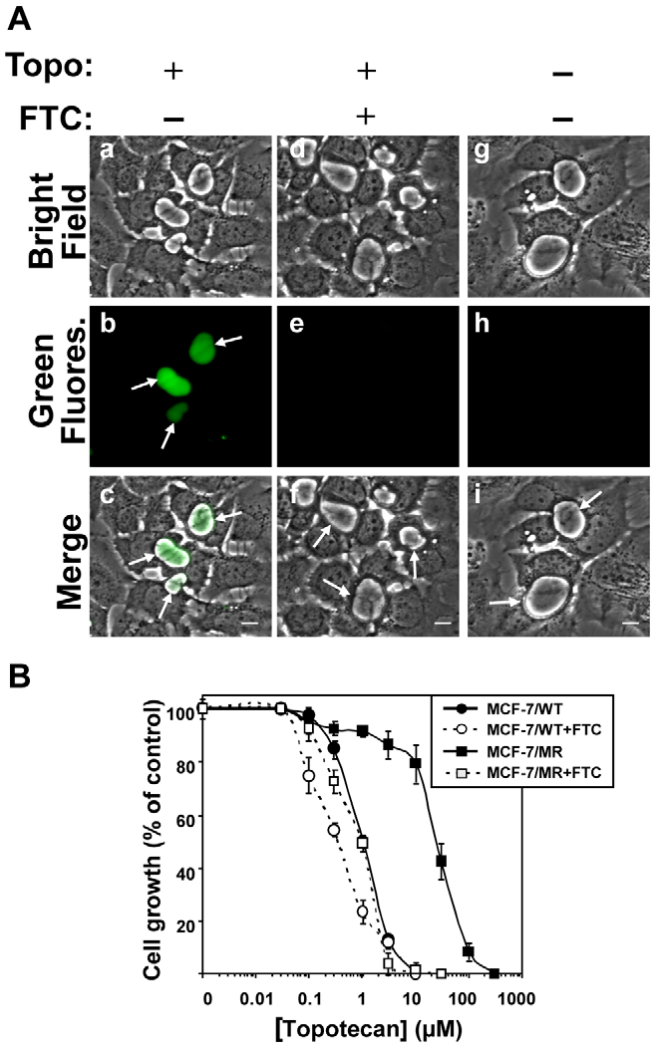
Inhibition of cell growth by topotecan and its accumulation in EVs. (A) MCF-7/MR cells were grown in riboflavin-deficient medium for 7 days on dishes containing cover glass bottom. Then, cells were incubated with topotecan (5µM) for 24hr at 37°C in the presence (d–f) or absence (a–c) of FTC (10µM). Control cells were cultured in drug-free and riboflavin-deficient medium (g–i). Intravesicular accumulation of topotecan was analyzed as described in Materials and Methods. *Arrows* indicate the localization of EVs that lack or contain topotecan. (B) MCF-7 and MCF-7/MR cells were grown for 3 days, exposed to various concentrations of topotecan for 72h in the absence or presence of FTC (10µM), following which the cytotoxic effect was determined by the colorimetric XTT assay. Shown are the means of three independent experiments, each performed in triplicates ± SD. Topotecan IC_50_ values in MCF-7 and MCF-7/MR cells were 1.04±0.06 and 25.5±2.6, respectively.

The ability of ABCG2-rich EVs to concentrate and sequester various anticancer drugs within the vesicular lumen was explored. Towards this end, we first examined the natural chromophoric anticancer drug topotecan that elicits its cytotoxic activity by stabilizing a covalent topoisomerase I-DNA complex, thereby inflicting a hindrance to the progression of DNA replication fork with subsequent formation of lethal DNA lesions [29]. Topotacan is an established ABCG2 substrate [30], [31], [32] and is currently approved for the treatment of various cancers. MCF-7/MR cells grown in a riboflavin-deficient medium to avoid its intense intravesicular green fluorescence [12], were incubated with topotecan (5µM) in the presence or absence of the specific ABCG2 transport inhibitor FTC. Taking advantage of its endogenous fluorescence, topotecan was found to highly accumulate in EVs in an ABCG2-dependent manner (**Figure 8A**). Consequently, MCF-7/MR cells displayed 25-fold resistance to topotecan, when compared to their parental MCF-7 cells; this resistance was fully reversed by the ABCG2 transport inhibitor FTC (**Figure 8B**). In addition to topotecan, we performed similar accumulation experiments using viable cell fluorescence microscopy studies with additional fluorescent compounds which are cytotoxic agents with distinct structure and mode of action (as well as non-toxic compounds including riboflavin); these included established ABCG2 substrates and non-ABCG2 substrates. The results of the accumulation in EVs are summarized in **Table 1**; whereas established chromophoric ABCG2 cytotoxic substrates accumulated in EVs, non-ABCG2 substrates failed to accumulate in EVs. These results highlight the remarkable capacity of ABCG2-rich EVs to markedly sequester various anticancer drugs.

**Table 1:** Multiple anticancer drug concentrative capacity of EVs.

Fluorescent compound		Concentration (µM)	Accumulation in EVs	Inhibition of accumulation in EVs^a^
Topotecan		5	+	+
Imidazoacridinones	C-1371	5	+	+
	C-1492	5	+	+
	C-1309	5	+	+
	C-1336	5	+	+
	C-1633	5	+	+
	C-1266	5	−	NS
Fluorescein-methotrexate		10	+	+
Hoechst 33342		10	+	+
Mitoxantrone		20	+	+
Riboflavin^b^		20	+	+

a-10µM FTC were used to block ABCG2-dependent drug transport.

b-Not cytotoxic.

NS-Not a substrate.

## Discussion

**Figure 9:**
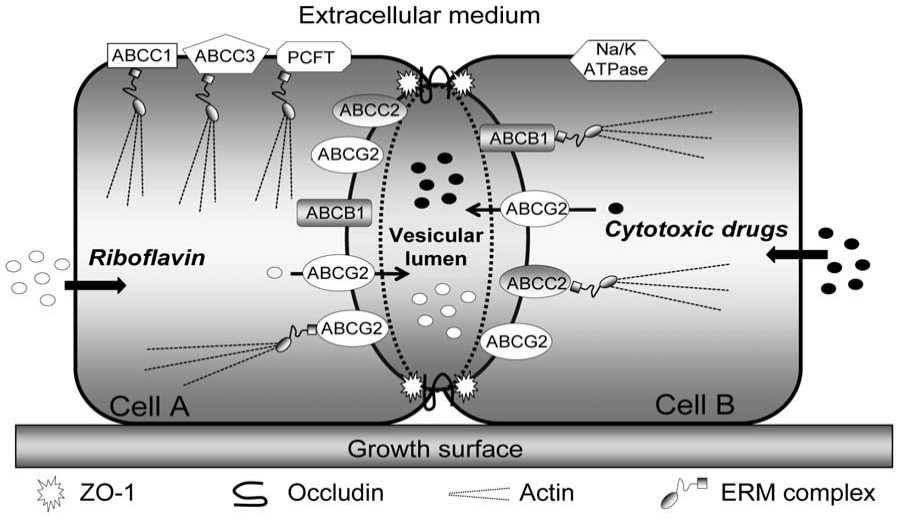
A proposed model of EVs structure and function. Shown is an EV formed between two attached MCF-7/MR cells. The EV is structurally reinforced by an actin cytoskeleton. TJ proteins including occludin and ZO-1 (indicated by the dashed line surrounding the vesicles) seal the EV to the extracellular milieu and to the surrounding cells. ABCG2 is highly expressed on the EV membrane, but not on the cell membrane facing the medium or neighbor cells. ABCG2-rich EVs highly concentrate multiple cytotoxic agents including topotecan, methotrexate, imidazoacridinones, Hoechst 33342 and MR [8] as well as the B2-vitamin riboflavin [12]. Just like ABCG2, ABCB1 and ABCC2 are also differentially targeted to the EV membrane, whereas ABCC1, ABCC3 and PCFT are localized to the cell membrane. Na^+^/K^+^ ATPase is present at the cell membrane. ERM proteins, which are highly targeted to the EV surface, presumably anchor these ABC transporters to the actin cytoskeleton.

Based on our present findings and on our previous studies with EVs in MCF-7/MR cells [8], [12], we propose a composite model for the structure and function of ABCG2-rich EVs, and their ability to confer resistance to multiple anticancer drugs (**Figure 9**). Two or more attached cells form a tightly sealed extracellular compartment termed EV. The oval-shape structure of this EV is reinforced by actin microfilament-based cytoskeleton, thus forming an actin-rich brush border at the apical side of MCF-7/MR cells. Moreover, as would be expected from a compartment that highly concentrates multiple chemotherapeutics, EVs are secluded from the extracellular milieu by TJ proteins including occludin and ZO-1, which localize at the border between EV-forming cells, in a belt-like pattern. Each EV-forming cell contributes its relative share of the vesicular structure; e.g. when three attached cells form a common EV, three distinct belt-like structures of TJ proteins are apparent. ABCG2 is overexpressed and selectively targeted to the EV membrane. This unique localization of ABCG2 mediates the efficient pumping and hence concentration of multiple cytotoxic agents of distinct structure and mode of action (as well as non toxic compounds including riboflavin) from the cytoplasm into the lumen of EVs. These cytotoxic agents include topotecan, imidazoacridinones, methotrexate, MR and Hoechst 33342, hence representing various families of anticancer drugs. Most importantly, we further discovered that apart from ABCG2, key MDR efflux transporters including ABCB1 and ABCC2 are also selectively targeted to the EV membrane. Moreover, this differential targeting to the EVs membrane cannot be considered a general default phenomenon for all transporters, since ABCC1, ABCC3 and PCFT were targeted to the cell membrane but not to the EVs membrane. Likewise, Na^+^/K^+^ ATPase was directed to the cell membrane. We also found here that ERM proteins displayed a remarkably differential sorting to, and high abundance in the EV membrane border, thereby suggesting a functional role for the anchoring of EV-targeted transporters to the actin cytoskeleton. Hence, the formation of sealed EVs shared by attached breast cancer cells and the specific targeting of a network of MDR efflux transporters to the EV membrane is a novel modality of anticancer drug compartmentalization and consequent MDR. In this respect, a variety of cytotoxic ABCG2 transport substrates that enter cells by diffusion or via carrier-mediated transport, are extracted from the cytoplasm and actively pumped into the vesicular lumen by ABCG2.

The current study suggested a structural and functional homology between the EVs of MCF-7/MR breast cancer cells and bile canaliculi. First, the localization pattern of TJ proteins observed in EVs was precisely that previously described for WIF-B9 [15] and HepG2 cells [16], both of which form bile canaliculi and target ABCB1, ABCC2, ABCG2 and ABCB11 (BSEP, bile salt excretory protein) to the canalicular membrane [33], [34], [35]. This unique localization of TJ proteins could possibly function as a gate which controls the passage of ions and solutes between the cytoplasm and the vesicular lumen, and as a fence which differentiates between transmembrane proteins of the apical and basolateral membranes. Thus, ABCB1, ABCC2 and ABCG2, which are the predominant characteristic transporters of polarized bile canaliculi and hepatocytes [36], [37], were consistently and selectively sorted to the EVs membrane. Second, just like polarized liver cells, MCF-7/MR cells displayed a polarized distribution of cytoskeletal elements; actin filaments were predominantly concentrated in a thin network around the EVs membrane, whereas microtubules radiated out from EVs. Thus, it is likely that microtubules are oriented with their minus end towards the apical domain (i.e. EVs), as found for other polarized epithelial cells including MDCK and WIF-B9 cells, and that they play a key role in polarized trafficking of transmembrane proteins [27]. Third, not only was the localization pattern of actin filaments and microtubules in MCF-7/MR cells similar to that reported for WIF-B9 and HepG2 cells, the impact of treatment with CytD and nocodazole, was found to be identical in MCF-7/MR cells and hepatoctyes [15], [16]. Forth, it is yet unclear how exactly actin filaments participate in apical trafficking, cycling and endocytosis of ABC transporters in the liver and secretory epithelia including breast epithelium. However, a study implicating actin participation in targeting and/or maintenance of apical localization of ABC transporters involves knockout mice in which the radixin gene, encoding for the dominant ERM protein in the liver, was eliminated by targeted disruption [38]; hence, elimination of radixin, which is localized at the bile canalicular membrane, resulted in progressive dilation of canaliculi, decreased microvilli and most importantly jaundice, due to impaired apical trafficking of ABCC2 and disappearance of other characteristic canalicular ABC transporters. Consistently here, ERM proteins perfectly co-localized with ABCG2 in the EVs membrane border and in cell-cell attachment zones of MCF-7/MR and parental MCF-7 cells. This is a novel observation as no direct interaction between ABCG2 and ERM proteins was reported to date [39]. We therefore propose that the possible interaction between structural proteins of the ERM complex and ABC transporters is essential for their proper localization, retention and function at the EVs membrane. Hence, EVs localization of ERM proteins may be a prerequisite for vesicular biogenesis and function of ABC transporters.

Ectopic overexpression of ABCB1 and ABCC2 in transfectant MCF-7/MR cells resulted in a unique pattern of hemi-vesicular distribution of these MDR pumps in the EV membrane. Specifically, immunofluorescence microscopy revealed that ABCB1 and ABCC2 localized in only one half of the EV membrane, whereas endogenous ABCG2 was equally distributed in the EV membrane. This important observation is in complete concordance with a previous study in which WIF-B9 cells were transfected with YFP-ABCB11, thereby resulting in the same staining pattern referred to as “hemi-canaliculi” [28]. Likewise, our present hemi-vesicular staining pattern of ABCB1 and ABCC2 could be explained by the fact that EVs are formed and shared by two (or more) attached cells, one of which was successfully transfected with the ABC transporter gene, whereas its neighbor counterpart was not, due to the relatively low transfection efficiency (∼10–15%). Thus, the EV membrane presumably originates from the plasma membrane where each attached cell contributes its relative share of the EV membrane and its transmembrane proteins.

The current study and our previous reports [8], [12] provide evidence supporting the conclusion of the gradual development and biogenesis of EVs from early and premature vesicular structures to mature EVs: 1) Previously we have shown that the membrane of both premature and mature EVs is a lipid bilayer with multiple microvilli-like invaginations protruding into the intravesicular lumen, which are likely to increase the vesicular membrane surface area, and hence transmembrane fluxes of cytotoxic agents [8]. 2) ZO-1 was observed precisely at the border between EVs-forming attached cells, even at the early stages of EVs formation, when ABCG2 was present only at cell-cell attachment zones. 3) ABCG2-rich crucifer-like structures forming at cell-cell attachment zones co-localized with ERM proteins and were readily capable of concentrating cytotoxic agents that are established ABCG2 substrates including topotecan, imidazoacridinones, methotrexate, MR, Hoechst 33342 and the B2-vitamin riboflavin just like mature EVs. These cumulative evidences indicate that premature EVs structures are indeed early EVs. Following ABCG2-dependent intravesicular concentration of various substrates, these early EVs structures fill up with fluids, hence leading to gradual increase in intravesicular volume, thereby resulting in the formation of mature oval-shaped EVs. Thus, in mature EVs, ABCG2 localizes specifically to the EVs membrane, whereas TJ proteins surround the EVs in a zipper-like manner precisely at the border of cells. This ABCG2 protein level-dependent biogenesis process explains the observed differences between the multiple, mature EVs observed in MCF-7/MR cells and the rare, small and irregularly shaped EVs of parental MCF-7 cells (as shown in **Figure 4**); this is in good agreement with the differential targeting of the ectopically overexpressed ABCG2-EGFP in parental MCF-7 and MCF-7/MR cells (**Supplementary Figure S2**).

The current study constitutes a novel modality of chemoresistance to multiple anticancer drugs mediated by EVs in breast cancer cells. The possible existence of such multidrug-concentrating compartments in tumor-derived specimens, allowing sequestration of anticancer drugs away from their intracellular pharmacological targets is underway in our laboratory, and may have important implications for the overcoming of MDR phenomena in cancer chemotherapy. Since ABCG2 transport inhibitors fully restored drug sensitivity to various anticancer drugs, this could be potentially applied in MDR tumors with ABCG2-rich EVs. Inhibition of EVs biogenesis could be an alternative approach; our preliminary studies using specific inhibitors of the PI3K-Akt signaling axis (e.g. LY294002), reveal a marked decrease in both the number of EVs and their volume (data not shown). ABCG2 associated with the EVs membrane is presumably retracted to the intracellular compartment, thereby resulting in EVs that are unable to concentrate endogenously fluorescent cytotoxic agents, thus restoring drug sensitivity in a dose- and time-dependent manner.

## Materials and Methods

### Chemicals

Cytochalasin D (CytD), nocodazole, Hoechst 33342, fumitremorgin C (FTC), MR, topotecan, riboflavin, DAB, 3′-amino 9′-ethyl carbazole (AEC), hematoxylin, Triton X-100 and DAPI were purchased from Sigma-Aldrich (St. Louis, MO). Rhodamine-phalloidin and fluorescein-methotrexate were from Invitrogen (Carlsbad, CA). Imidazoacridinones were synthesized by Prof. M. Cholody, B. Horowska and M. Konieczny and kindly provided by Prof. A. Skladanowski, Gdansk University, Gdansk, Poland.

### Tissue culture

Human breast cancer MCF-7 cells, their MR-resistant subline MCF-7/MR as well as flavopiridol-resistant MCF-7/FLV1000 cells were grown as described previously [8]. Prior to vesicular drug accumulation experiments, cells were grown in custom-made riboflavin-deficient RPMI-1640 medium (Biological Industries, Beth-Haemek, Israel) supplemented with 10% dialyzed fetal calf serum (Invitrogen, Carlsbad, CA), glutamine and antibiotics.

### Immunohistochemistry

MCF-7/MR cells (2×10^4^cell/2ml) were seeded in 24-well plates and incubated for 4 days at 37°C. Immunohistochemical analysis with anti-ABCG2 monoclonal antibody BXP-53 (1∶100 dilution) or with a polyclonal antibody directed to the β-subunit of Na^+^/K^+^ ATPase (GERK; 1∶100 dilution) was performed as described previously [12]. Color development was then carried out using either 0.6 mg/ml DAB or 0.4mg/ml AEC in a buffer solution containing 0.1M sodium acetate pH 4.7 and 0.02% H_2_O_2_. After counterstaining of nuclei with hematoxylin, cells were examined with a Leica inverted microscope using a bright field mode.

### Immunofluorescence studies

Cells were seeded (5×10^3^ cells/2ml) in 24-well plates on sterile glass coverslips and incubated for 7 days at 37°C. Cells were processed with an identical staining protocol as described above (see *Immunohistochemistry microscopy*) with the following modifications: first, BXP-21 served as the primary ABCG2-specific monoclonal antibody. TJ proteins were visualized using either rabbit-anti-occludin antibody or a mouse anti-ZO-1 monoclonal antibody (1∶25, Invitrogen, Carlsbad, CA). The ERM protein complex was visualized using rabbit monoclonal anti-ERM antibody (1∶500 dilution, Epitomics, Burlingame, CA), which detects all three ERM proteins. Second, FITC-conjugated donkey anti-mouse IgG or rhodamine red-conjugated donkey anti-rabbit antibody (1∶100 dilution, Jackson ImmunoResearch Laboratories, West Grove, PA) served as the secondary antibodies. Third, cell nuclei were counterstained with the DNA dye DAPI (0.5µg/ml) during the incubation with the secondary antibody. Finally, for F-actin and β-tubulin staining, cells were permeabilized with 0.1% Triton-X-100 in PBS for 10 min and immunoreacted as described above. To visualize F-actin, rhodamine phalloidin was added during the last 20 min of incubation with the secondary antibody. Microtubules were followed using mouse monoclonal antibody to β-tubulin (at 1∶200 dilution, Sigma). After four washes with PBS, coverslips were mounted onto glass slides using Fluoromount-G (Southern Biotechnology Associates, Birmingham, AL) and examined using either a Leica inverted or Zeiss inverted Cell-Observer or a confocal Zeiss LSM 510 META microscope. Merged images were obtained using either the Leica software or the AxioVision program (Zeiss, version 4.7).

### Live cell imaging

MCF-7/MR cells were seeded in 24-wells plates on sterile glass coverslips (5×10^3^cells/2ml) or in dishes containing cover glass bottom (3×10^4^cells/2ml; World Precision Instruments) and grown in riboflavin-containing or -lacking RPMI-1640 medium for at least 72 hr prior to drug addition. Cells were then incubated either with CytD (10µg/ml) for 30 min or with various chromophoric cytotoxic drugs (Table 1) for 24 hr at 37°C. In all live imaging microscopy experiments, cells were washed thrice with PBS, resuspended in PBS supplemented with 1mM CaCl_2_, 1mM MgCl_2_ and 10mM D-glucose and random colonies were analyzed using Zeiss inverted Cell-Observer microscope using the following filters: phase mode, HE GFP (excitation and emission at 470 and 525 nm, respectively) or DAPI mode (excitation and emission at 365 and 445 nm, respectively) at a magnification of ×200–×630.

### Subcellular localization of MDR transporters and PCFT (SLC46A1)

MCF-7/MR cells were grown in 24-well plates containing glass coverslips. Following 4 days of growth, monolayer cells were transiently transfected using the jetPEI transfection reagent (Polyplus-transfection Inc. New York, USA), according to the manufacturer's instructions. The following constructs were used: pcDNA3 harboring ABCC1, ABCC2 or ABCC3; pcDNA3.1 harboring Myc-tagged PCFT [23] and pHAMDR1/WT construct (AddGene). Forty to forty eight hr after transfection, cells were fixed with 4% formaldehyde for 20 min and reacted with specific antibodies as described in *Immunofluorescence Studies*. In addition, cells were stably transfected with a pcDNA3 harboring ABCC2 or pEGFP-ABCG2 N1 construct (kindly provided by Prof. L. Homolya) [40], using the same protocol and grown continuously in selective medium containing 1mg/ml G418.

### Immunofluorescence microscopy studies exploring the polarity of MCF-7/MR cells

MCF-7/MR cells were grown and transiently transfected with GPI-CFP and VSVG-YFP constructs (generously provided by Prof. D. Cassel, Technion, Israel). To visualize VSVG, following transfection, cells were incubated for 18h at 40°C and transferred to 32°C for 2h prior to fixation. ABCG2 was visualized using BXP-53 antibody. Cells were then analyzed using Zeiss inverted Cell-Observer.

### Western blot analysis

ABCG2 and ERM protein levels, as normalized to Na^+^/K^+^-ATPase, were determined by Western blot using anti-ABCG2 (BXP-53; 1∶3,000 dilution), anti-ERM (at 1∶500 dilution) and anti-Na^+^/K^+^-ATPase (KETTY at 1∶3000 dilution) antibodies, respectively, as described previously [8].

#### Cytotoxicity assay

Four to five hundred parental MCF-7 and MCF7/MR cells were seeded (90µl/well) in 96-well plates, in a riboflavin-deficient medium and grown for 3 days to allow for the formation of EVs. Cells were then subjected to increasing concentrations of topotecan and incubated for 72h in the absence or presence of the ABCG2 transport inhibitor FTC (10 µM). The cytotoxic effect was determined using a colorimetric XTT cell proliferation kit (Biological Industries, Beth-Haemek, Israel).

### Flow cytometric analysis of surface ABCG2 expression in viable cells

One million cells were washed thrice in PBS and suspended in 100µl PBS containing 1% BSA. Then, cells were incubated either with or without a PE-conjugated anti-human ABCG2 monoclonal antibody 5D3 (0.25µg/100µl; eBioscience, San Diego, CA). After 30 min of incubation at 37°C, cells were transferred to ice-cold water, washed thrice with PBS, resuspended in ice-cold PBS containing 1% BSA and cellular fluorescence was determined using a FACSCalibur flow cytometer (BD Bioscience). FL2-H excitation of PE-labeled cells was performed at 550nm and emission was collected at 574nm. Data analysis was performed using FCSexpress software.

## Supporting Information

Figure S1 **ABCG2 specifically localizes to the EVs membrane in MCF-7 cells.** MCF-7 cells were grown and analyzed by immunofluorescence microscopy as described in Figure 1 legend. ABCG2 (red fluorescence), ZO-1 (green fluorescence), nuclei (blue fluorescence). *Arrows* denote the location of the EVs.
(TIF)

Figure S2 **EGFP-ABCG2 overexpression in transfectant MCF-7 and MCF-7/MR cells results in its differential targeting.** MCF-7 (A–C) and MCF-7/MR cells (D–F) were stably transfected with the pEGFP-ABCG2 N1 construct as described in Materials and Methods, grown in riboflavin-deficient medium for at least 4 days prior to fluorescence microscopy and studied using a Leica microscope (×200). *Arrows* denote the location of ABCG2-EGFP either in ABCG2-rich EVs (D–F) or at the cell membrane (A–C). Untransfected MCF-7/MR cells (G–H) were grown and analyzed by immunofluorescence as in Figure 1. *Continuous arrows* denote the location ABCG2 in cells that do not form EVs. Note that ABCG2 in the EVs-forming cells is predominantly localized to the EVs (*dashed arrow*), with no residual signal in ER or cell membrane.
(TIF)

Figure S3 **Localization of endogenous Na^+^/K^+^ ATPase in MCF-7/MR cells.** *Immunohistochemistry:* MCF-7/MR cells were reacted either with BXP-53 (A and D) or with anti-β subunit of Na^+^/K^+^ ATPase polyclonal antibody (GERK; panels B and E), followed by HRP-conjugated goat anti-rat or anti-rabbit IgG, respectively, and color development was carried out using either the red chromogen AEC (A–C) or the brown chromogen DAB (D–F). As a control, cells were reacted solely with secondary antibodies including HRP-conjugated goat anti-rabbit (C) or anti-rat (F). Cells were then examined using a Leica microscope at ×400 magnification at a bright field mode. The *continuous arrows* denote the location of EVs, whereas the *dashed arrows* point to the cell membrane containing Na^+^/K^+^ ATPase but not ABCG2. *Immunofluorescence:* MCF-7/MR cells were reacted either with BXP-21 (G) or with an anti-Na^+^/K^+^ ATPase antibody (H). Nuclei were counterstained with DAPI. Stained cells were then analyzed using a laser scanning confocal microscope (Zeiss LSM 510 META). A merged image (I) was obtained using the Zeiss LSM software. Na^+^/K^+^ ATPase localization to the membrane of EVs was examined by performing Z-stack sections creating 15 optical slices (0.5µm thick each). Z-sectioning images of the indicated area in I (*white square*) is shown in panel J, where the horizontal and the vertical lines indicate the exact position of the Z-stack.
(TIF)

Figure S4 **Large Gaussian distribution of surface ABCG2 expression in MCF-7/MR and A549/K1.5 cells.** Live MCF-7/MR and A549/K1.5 cells were incubated either with (B and D, respectively) or without (A and C, respectively) PE-conjugated anti-human ABCG2 antibody (5D3) as described in Materials and Methods. Shown are representative results summarized as dot graphs. Y- axis presents the side scatter count, whereas the X-axis represents ABCG2 fluorescence. Geometric means of MCF-7/MR and A549/K1.5 cell were estimated as 331.8±15.0 and 689.4±46.2, respectively, whereas geometric means of auto-fluorescence were approximately 2.0±0.2 in both cell lines.
(TIF)

Video S1 **Localization of ABCG2 and F-actin in MCF-7/MR cells.** Cells were grown for 7 days, fixed with 4% formaldehyde and permeabilized using Triton-X-100. Then, cells were reacted with anti-ABCG2 antibody (BXP-21, green fluorescence) as well as with rhodamine phalloidin which binds to actin filaments (red fluorescence). Nuclei were stained with DAPI (blue fluorescence). Slides were analyzed by Z-stack sections creating 12 optical slices (0.5µm thick each) using a laser-scanning confocal microscope (Zeiss LSM 510 META) at ×630 magnification. The merged image of all obtained slices was animated into a video using LSM image browser software (Zeiss Inc.).
(AVI)

Video S2 **Localization of ABCG2 and Na^+^/K^+^ ATPase in MCF-7/MR cells.** Cells were grown for 7 days, fixed with 4% formaldehyde and reacted with anti-ABCG2 antibody (BXP-21, green fluorescence) as well as with an anti-Na^+^/K^+^ ATPase antibody (GERK, red fluorescence). Cells were then analyzed by Z-stack sections creating 15 optical slices (0.5µm thick each) using a laser-scanning confocal microscope (Zeiss LSM 510 META) at a magnification of ×630. The merged image of all obtained slices was animated into a video using LSM image browser software (Zeiss Inc.).
(AVI)
