# Supplementary figures and images for: Structure and Function of ABCG2-Rich Extracellular Vesicles Mediating Multidrug Resistance
Source: PLoS One. 2011 Jan 24;6(1):e16007. doi: 10.1371/journal.pone.0016007 (PMC3025911; doi:10.1371/journal.pone.0016007)

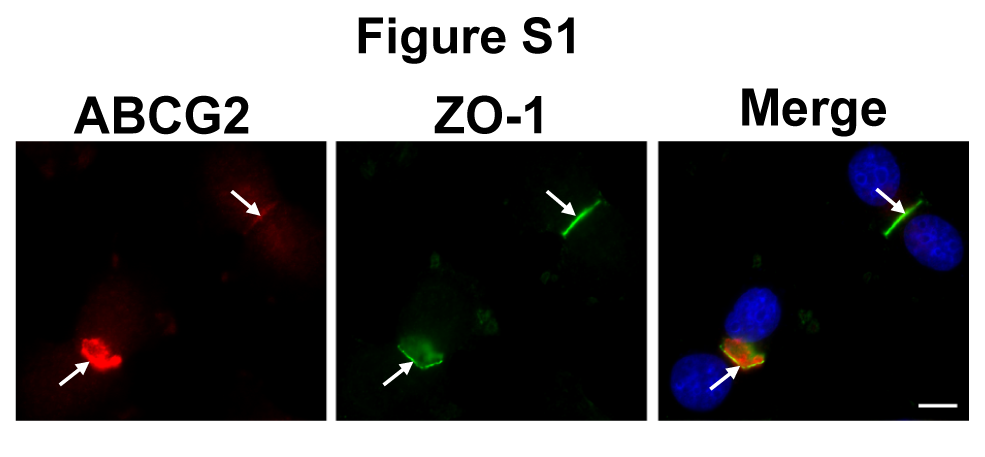

Supplement: Figure S1 — ABCG2 specifically localizes to the EVs membrane in MCF-7 cells. MCF-7 cells were grown and analyzed by immunofluorescence microscopy as described in Figure 1 legend. ABCG2 (red fluorescence), ZO-1 (green fluorescence), nuclei (blue fluorescence). Arrows denote the location of the EVs. (TIF) [file pone.0016007.s001.tif]

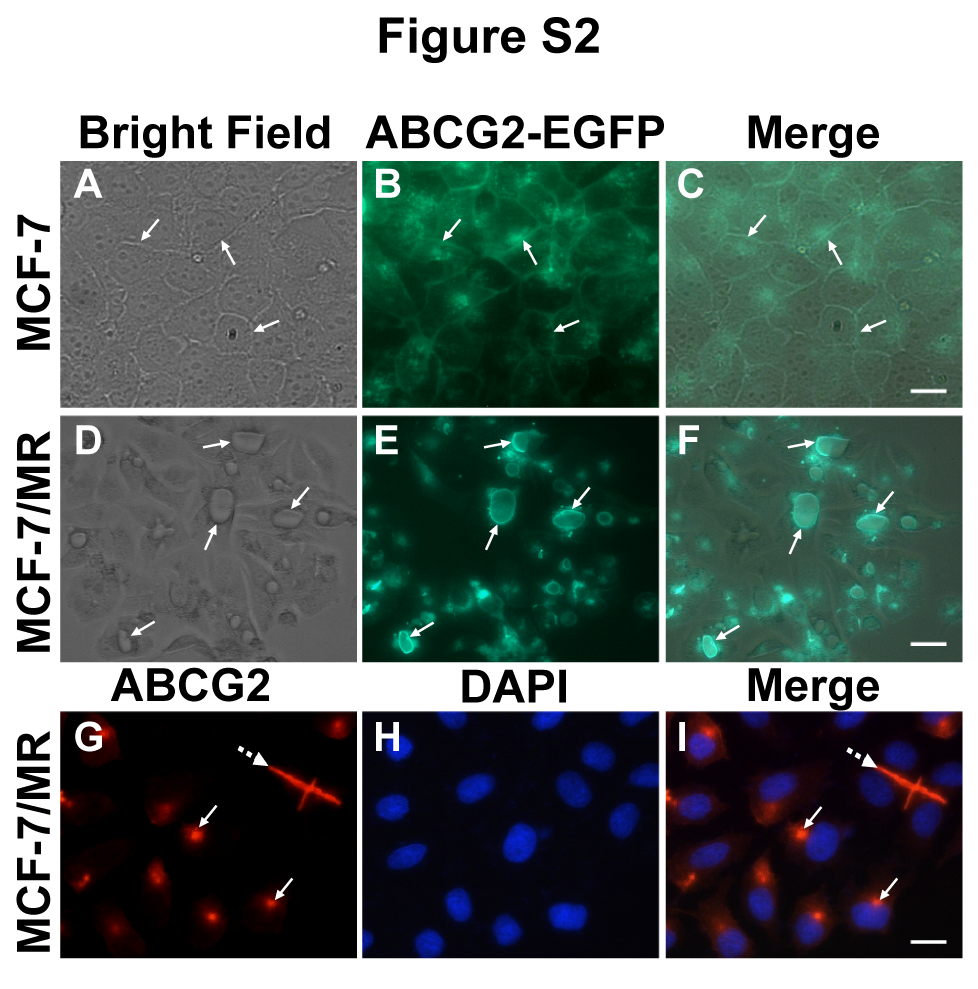

Supplement: Figure S2 — EGFP-ABCG2 overexpression in transfectant MCF-7 and MCF-7/MR cells results in its differential targeting. MCF-7 (A–C) and MCF-7/MR cells (D–F) were stably transfected with the pEGFP-ABCG2 N1 construct as described in Materials and Methods, grown in riboflavin-deficient medium for at least 4 days prior to fluorescence microscopy and studied using a Leica microscope (×200). Arrows denote the location of ABCG2-EGFP either in ABCG2-rich EVs (D–F) or at the cell membrane (A–C). Untransfected MCF-7/MR cells (G–H) were grown and analyzed by immunofluorescence as in Figure 1. Continuous arrows denote the location ABCG2 in cells that do not form EVs. Note that ABCG2 in the EVs-forming cells is predominantly localized to the EVs (dashed arrow), with no residual signal in ER or cell membrane. (TIF) [file pone.0016007.s002.tif]

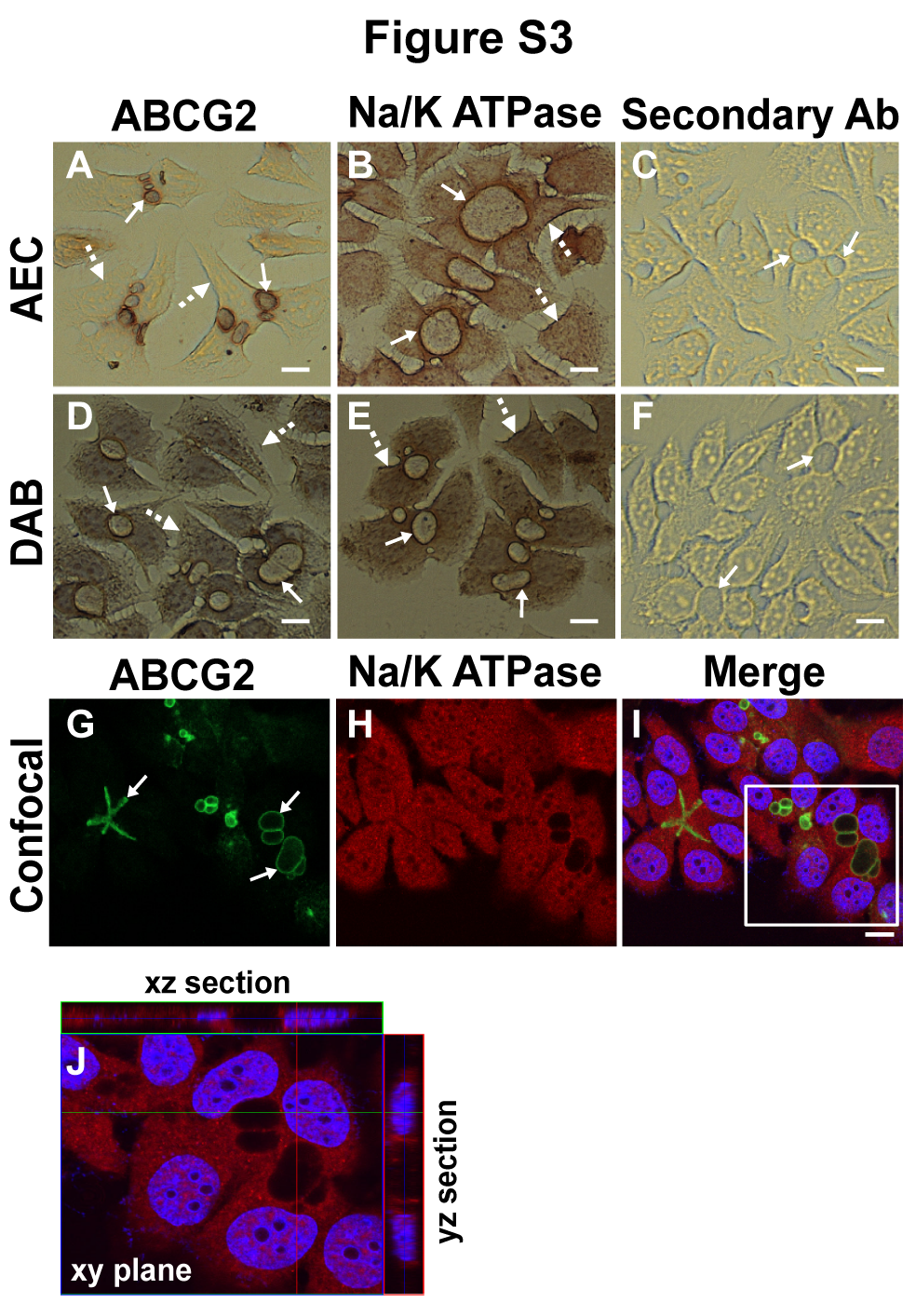

Supplement: Figure S3 — Localization of endogenous Na+/K+ ATPase in MCF-7/MR cells. Immunohistochemistry: MCF-7/MR cells were reacted either with BXP-53 (A and D) or with anti-β subunit of Na+/K+ ATPase polyclonal antibody (GERK; panels B and E), followed by HRP-conjugated goat anti-rat or anti-rabbit IgG, respectively, and color development was carried out using either the red chromogen AEC (A–C) or the brown chromogen DAB (D–F). As a control, cells were reacted solely with secondary antibodies including HRP-conjugated goat anti-rabbit (C) or anti-rat (F). Cells were then examined using a Leica microscope at ×400 magnification at a bright field mode. The continuous arrows denote the location of EVs, whereas the dashed arrows point to the cell membrane containing Na+/K+ ATPase but not ABCG2. Immunofluorescence: MCF-7/MR cells were reacted either with BXP-21 (G) or with an anti-Na+/K+ ATPase antibody (H). Nuclei were counterstained with DAPI. Stained cells were then analyzed using a laser scanning confocal microscope (Zeiss LSM 510 META). A merged image (I) was obtained using the Zeiss LSM software. Na+/K+ ATPase localization to the membrane of EVs was examined by performing Z-stack sections creating 15 optical slices (0.5µm thick each). Z-sectioning images of the indicated area in I (white square) is shown in panel J, where the horizontal and the vertical lines indicate the exact position of the Z-stack. (TIF) [file pone.0016007.s003.tif]

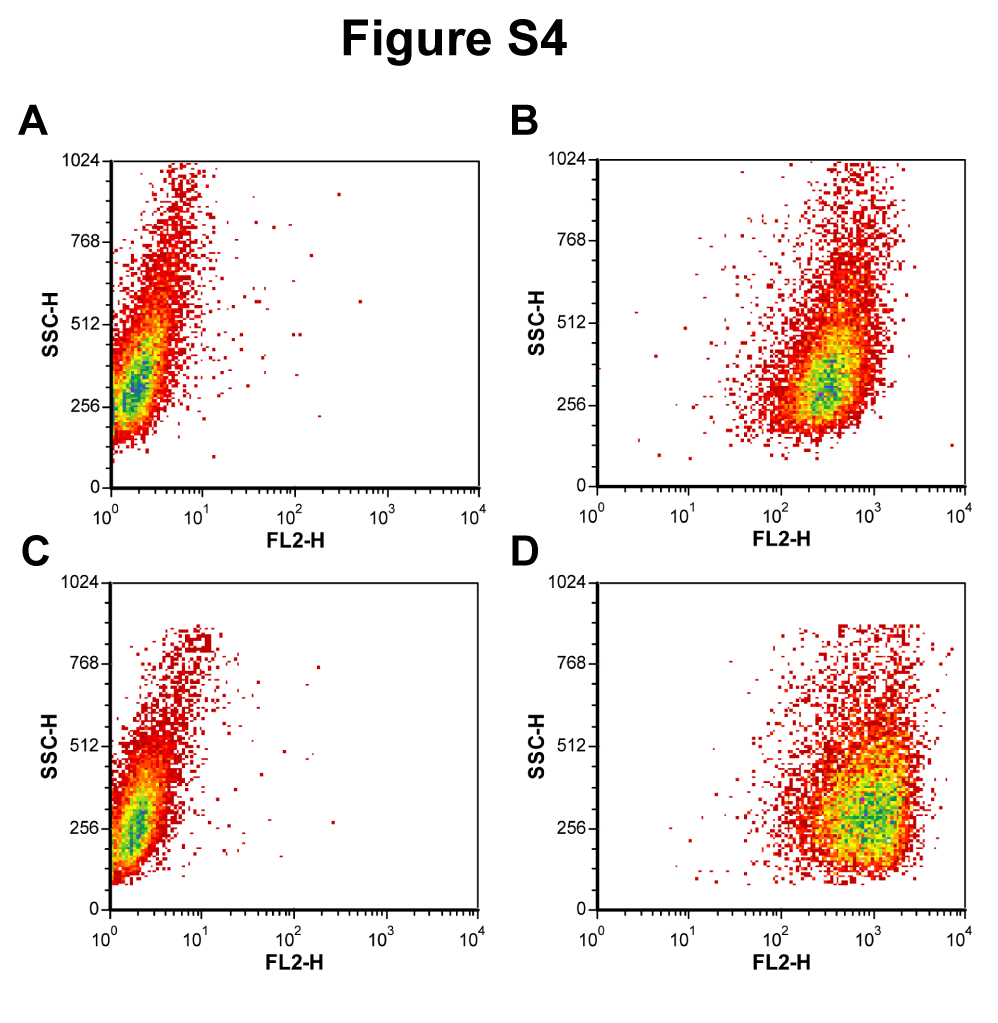

Supplement: Figure S4 — Large Gaussian distribution of surface ABCG2 expression in MCF-7/MR and A549/K1.5 cells. Live MCF-7/MR and A549/K1.5 cells were incubated either with (B and D, respectively) or without (A and C, respectively) PE-conjugated anti-human ABCG2 antibody (5D3) as described in Materials and Methods. Shown are representative results summarized as dot graphs. Y- axis presents the side scatter count, whereas the X-axis represents ABCG2 fluorescence. Geometric means of MCF-7/MR and A549/K1.5 cell were estimated as 331.8±15.0 and 689.4±46.2, respectively, whereas geometric means of auto-fluorescence were approximately 2.0±0.2 in both cell lines. (TIF) [file pone.0016007.s004.tif]
